# Automated grading of chest x-ray images for viral pneumonia with convolutional neural networks ensemble and region of interest localization

**DOI:** 10.1371/journal.pone.0280352

**Published:** 2023-01-17

**Authors:** Asad Khan, Muhammad Usman Akram, Sajid Nazir

**Affiliations:** 1 Computer and Software Engineering Department, National University of Sciences and Technology, Islamabad, Pakistan; 2 Department of Computing, Glasgow Caledonian University, Glasgow, United Kingdom; Taipei Medical University, TAIWAN

## Abstract

Following its initial identification on December 31, 2019, COVID-19 quickly spread around the world as a pandemic claiming more than six million lives. An early diagnosis with appropriate intervention can help prevent deaths and serious illness as the distinguishing symptoms that set COVID-19 apart from pneumonia and influenza frequently don’t show up until after the patient has already suffered significant damage. A chest X-ray (CXR), one of many imaging modalities that are useful for detection and one of the most used, offers a non-invasive method of detection. The CXR image analysis can also reveal additional disorders, such as pneumonia, which show up as anomalies in the lungs. Thus these CXRs can be used for automated grading aiding the doctors in making a better diagnosis. In order to classify a CXR image into the Negative for Pneumonia, Typical, Indeterminate, and Atypical, we used the publicly available CXR image competition dataset SIIM-FISABIO-RSNA COVID-19 from Kaggle. The suggested architecture employed an ensemble of EfficientNetv2-L for classification, which was trained via transfer learning from the initialised weights of ImageNet21K on various subsets of data (Code for the proposed methodology is available at: https://github.com/asadkhan1221/siim-covid19.git). To identify and localise opacities, an ensemble of YOLO was combined using Weighted Boxes Fusion (WBF). Significant generalisability gains were made possible by the suggested technique’s addition of classification auxiliary heads to the CNN backbone. The suggested method improved further by utilising test time augmentation for both classifiers and localizers. The results for Mean Average Precision score show that the proposed deep learning model achieves 0.617 and 0.609 on public and private sets respectively and these are comparable to other techniques for the Kaggle dataset.

## Introduction

Coronavirus is a zoonotic pathogen that can cause kidney failure, respiratory complications, and pneumonia by infecting the human airways cells [1]. It has a fatality rate of around 2% [2]. Every aspect of life has been disrupted by COVID-19 over the world. Protective measures and early detection can boost the odds of survival as it is unlikely to abate soon. There have been 532,213,989 Coronavirus cases and 6,312,111deaths worldwide by 30 May 2022 [3]. This has resulted in enormous pressure on the already constrained healthcare establishments, healthcare workers and radiologists [4]. Identification of the infection in a timely fashion can increase the number of recovered cases.

Pneumonia can be viral, bacterial or fungal, and the patient has difficulty in breathing due to inflammation of lungs’ air sacs being filled with fluid [5]. Similarly, early detection of pneumonia can reduce the mortality rate [5]. Reverse Transcription Polymerase Chain Reaction (RT-PCR) is the recommended test by the World Health Organization (WHO) but is time-consuming, inconvenient and insufficient to diagnose COVID-19 [6–8]. One of the best-known methods for diagnosing the onset of COVID-19 is through the use of Chest X-Ray (CXR) images. This method is quicker and more reliable for diagnosis [9]. Compared to other image modalities such as Computed Tomography (CT) that are also used for COVID-19 diagnosis, CXR images are widely available in healthcare institutions [1, 7] and portable CXR systems are also available [8].

COVID-19 pandemic has overwhelmed the radiologists because they have to deal with the unprecedented challenges of diagnosing a significantly large number of CXR images [7]. A robust diagnosis system able to work on CXR can help alleviate some of these problems. The advantage of automatic detection comes primarily in the form of reducing the exposure of healthcare staff to the disease [2]. In addition, automated detection and diagnosis systems can aid the healthcare workforce in making a better decision regarding the level of care needed by a patient. A review of studies from Dec 2019 to Apr 2021 concluded that Support Vector Machines (SVM) and Convolutional Neural Networks (CNNs) were the most widely used automatic classification models [10]. The performance of CNN models with enough training examples can achieve human-level performance [11]. One way of coping with the additional load is to automate the disease diagnosis using the medical images as most of the best performing models in Computer Vision competitions are based on CNN [11–13].

An advantage that deep learning models have over the earlier machine learning techniques is that these can automatically infer the significant features [4, 12]. One of the most common applications of deep learning is in automating medical image analysis [4]. The diagnosis using images with deep learning models can provide performance that is at par with expert radiologists. This performance can be attributed to the amount of large-scale image data that has become available over time and the improved architectures of the deep learning models. However, if the data is not representative of the problem domain then the results can be underwhelming. Nevertheless, the deep learning methods do have challenges of their own. In case of medical images, some of the problems faced are that the image resolution is quite high, the labelled data is not frequently available and the data is not available in sufficient quantities.

The models have been improving constantly and one of the recent models that has produced better results than the earlier models is the EfficientNet [14]. This architecture has had many variations since, from B0 to B7 with accompanied higher accuracy and more parameters [15]. Recently, the second generation of [14] has been proposed with its own variants which have cut down on parameter inefficiency and training time [16].

Infection localisation in COVID-19 CXR images is required in addition to detection [17]. Using the localised opacities, the doctors can track the progression of the disease for the patient. Infection maps were proposed for localisation and severity grading of COVID-19 in CXR images by annotating the segmentation masks using a human-machine approach [18]. Naïve Bayes was used as meta learner with an ensemble consisting of four CNN classifiers achieving F1-score of 100, 98 and 98 for COVID-19, normal and pneumonia classes respectively [19]. The study used Generative adversarial network (GAN) architectures for synthetic image generation and Gradient-weighted Class Activation Mapping (Grad-CAM) [20] visualisations for interpretability [19].

In this paper, we propose to classify images into four categories: Negative for pneumonia, Typical, Indeterminate, and Atypical—using an ensemble of CNN models. Additionally, we find opacities in the CXR utilising object localisation architectures, which can give the radiologist more insight than a single output classification label. Although some studies use CT images [21–25] for detection, our work on COVID-19 detection will mainly cover only classification using CXR images.

## Related work

Due to the availability of large-scale datasets and greater computational resources, medical image diagnosis has shifted from classical machine learning techniques with handcrafted features to deep learning and specifically CNNs. This is why the recent focus on diagnosis using CXRs has shifted to CNN as well.

Medical image analysis typically involves detection of lesions which are then classified [12]. A total of six neural network models, with four pre-trained models (VGG16, VGG19, ResNet50, Inception-v3), and two models consisting of two and three convolutional layers, were used for binary classification of CXR images for pneumonia [5]. The researchers found out that model 2 and VGG network had the best performance among all six models with a recall of 98% and 95%, and F1 scores of 94% and 91% respectively [5]. Following a similar approach [26], also used five pre-trained CNN models (ResNet50, ResNet101, ResNet152, InceptionV3 and Inception-ResNetV2) for three different binary classifications with four classes: COVID-19, normal, viral and bacterial pneumonia. The pre-trained ResNet50 provided the highest accuracy for the three datasets [26]. In addition to CNNs, Capsule Networks were used for identifying COVID-19 in CXR images by [27]. Their models achieved an accuracy of 98.02% on 1019 images from four datasets containing images as normal, COVID-19 and Pneumonia. In addition, the researchers also worked on a cloud-based application for faster computation. Using CXR, a classification network called DFFCNet was proposed for COVID-19 diagnosis. The model utilised the EfficientNetV2 backbone network for feature extraction. The suggested framework outperformed the other selected models in experiments [28].

Some studies have used the combination of CXR and CT images for improving the classification performance [29, 30]. Pre-trained models like Xception, InceptionV3, and EfficientNetV2 were used to identify COVID-19 in CXR and CT images. For the CXR dataset, EfficientNetV2 with fine tuning performed the best, but the LightEfficientNetV2 model performed the best for the CT data set [31]. In another study, a multi-classification model was proposed for four classes (normal, COVID-19, Pneumonia, and lung cancer) by combining CXR and CT images. The study used VGG19+CNN, ResNet152, ResNet152V2+Gated Recurrent Unit (GRU), and ResNet152V2 + Bidirectional GRU and achieved the best scores with VGG19+CNN model with a 98.05% accuracy.

Monshi et al. [32] worked on data augmentation and hyperparameter optimisation for improving the results of multiclass classification (normal, pneumonia, and COVID-19). The proposed optimisations increased the VGG-19 and ResNet-50 accuracy by 11.93% and 4.97% respectively. EfficientNet-B0 [14] was found to achieve best results based on accuracy, precision, recall and F1-scores compared to other network architectures [32]. Data augmentation used translation (±10%), intensity shift (±10%), zoom (±15%), horizontal flip (±10%), and rotation (±10%) [8].

Instead of relying on a single CNN classifier for final output, methods that rely on an ensemble of several classifiers have also been proposed. Bhardwaj & Kaur [33] came up with an ensemble approach comprising Inceptionv3, DenseNet121, Xception, InceptionResNetv2 for classification of COVID-19, Pneumonia, and normal CXR images. They were able to achieve 98.33% and 92.36% accuracy for binary and multiclass classification respectively [33]. Similarly, a study compared 16 classifiers for COVID-19 in CXR images (COVID-19, normal, viral Pneumonia) and different ensemble classification techniques, determining that majority voting technique yields an accuracy of 99.314% [34].

A transfer learning approach was used for avoiding over and under fitting [35]. VGG16 model pre-trained on ImageNet Large Scale Visual Recognition Challenge (ILSVRC) weights was used. VGG16 has over 138 million trainable parameters with six blocks of 13 convolutions, five max pooling, and three fully connected layers [35]. The model was fine-tuned with CXR images [35]. The image dataset had 8474 CXR images, and the model classified the images into normal, pneumonia, and COVID-19 classes. The results without data augmentation were significantly lower compared to the results with data augmentation. This model achieved a COVID-19 detection sensitivity of 98.4%, and a three-class accuracy of 94.5% [35].

Even though feature selection is an inherent part of a CNN architecture, manual feature selection can still be applied. The CNN thus functions as a deep feature extractor. Using three CNN models, ResNet50, ResNet101, and InceptionResNetv2 were for feature extraction, followed by feature selection using particle Swarm Optimisation (PSO) and Ant Colony Optimisation (ACO), CXR images were classified into normal, pneumonia and COVID-19 classes with K Nearest Neighbours (kNN) and SVM in a framework proposed by [7]. The study used CXR images from Kaggle dataset comprising 219 COVID-19, 1341 Normal, and 1345 pneumonia images. An accuracy of 99.86% and F1 score of 99.08 with 10-fold cross validation were obtained [7]. LeNet-5 was used as a feature extractor, followed by classification using Extreme Learning Machines (ELM) using Chimp Optimisation Algorithm (ChOA) for improving the results [13]. The training and testing time for 3100 images with ChOA-ELM was 0.9474 and 2.937 secs respectively. COVID-Xray-5k and COVIDectioNet datasets were used and an accuracy of 98.25% and 99.11% respectively were obtained [13]. Ismael & Sengur [36] used deep feature extraction with pre-trained Resnet18, ResNet50, ResNet101, VGG16 and VGG19 was used for classification with SVM with different kernels. The binary classification used a dataset comprised of 200 normal and 180 COVID-19 CXR images. The combination of ResNet50 and SVM classifier with a Linear kernel had the best results with an accuracy of 94.7% [36].

Severity assessment of COVID-19 can help fight this highly contagious disease. Keeping this in view, the severity assessment of COVID-19 CXR images into mild, moderate, severe, and critical with CNN was proposed by [37]. The study utilised nine publicly available CXR datasets with 3260 images in total. The disease severity score was based on an opacity score by two radiologists. The CNN model comprised of 16 weighted layers. The hyperparameters were grouped as architectural and fine adjustment categories and the results of the proposed architecture were better compared to ResNet-101, AlexNet, VGG-16 etc. [37]. A method for lung segmentation and COVID-19 localisation was proposed using U-Net, U-Net++ and Feature Pyramid Networks (FPN) with ground truth lung segmentation by human-machine collaborative approach [38]. The proposed approach achieved sensitivity and specificity values above 99% for COVID-19 detection. Transformers have also recently been employed for classification and opacity-based severity grading. [39] used a large CXR dataset to train the backbone model so that it may learn low level generalised features, which were then used with a vision transformer based framework for COVID-19 diagnosis and severity quantification in a multitask learning method. The vision transformer and severity map were combined with the deep features from the backbone model for the prediction of disease class and severity quantification.

Even though, many techniques and frameworks have been proposed for the classification of different lung diseases and opacity localisation, there is a lack of a single framework that not only classifies a CXR image in a particular disease class but also segments the opacity regions on the lungs if the lungs are diseased. Furthermore, while pre-existing architectures have been experimented with in terms of different weight initializations and hyperparameter optimization, in order to cater for the low number of COVID associated pneumonia images–as is usually the case–classification auxiliary heads have not been used to improve the performance of the base network.

Keeping the above research gaps in view, the main contributions of this paper are summarised as follows:

- A single framework consisting of an ensemble of EfficientNetv2-L for classification trained on different subsets of data using transfer learning along with an ensemble of YOLOv5 [40] for localisation of opacity is proposed.
- Modification of EfficienetNetv2-L by introduction of classification auxiliary heads to the CNN backbone is presented.
- The proposed framework further uses test time augmentation, for both classifiers and localisers, resulting in improvement in results.
- It also introduces use of pseudo colour processing for opacity localisation using YOLOv5.

## Materials and methods

This section describes the dataset, image pre-processing and augmentation techniques, proposed CNN model and the application architecture.

### SIIM-FISABIO-RSNA COVID-19 detection dataset

**Fig 1:**
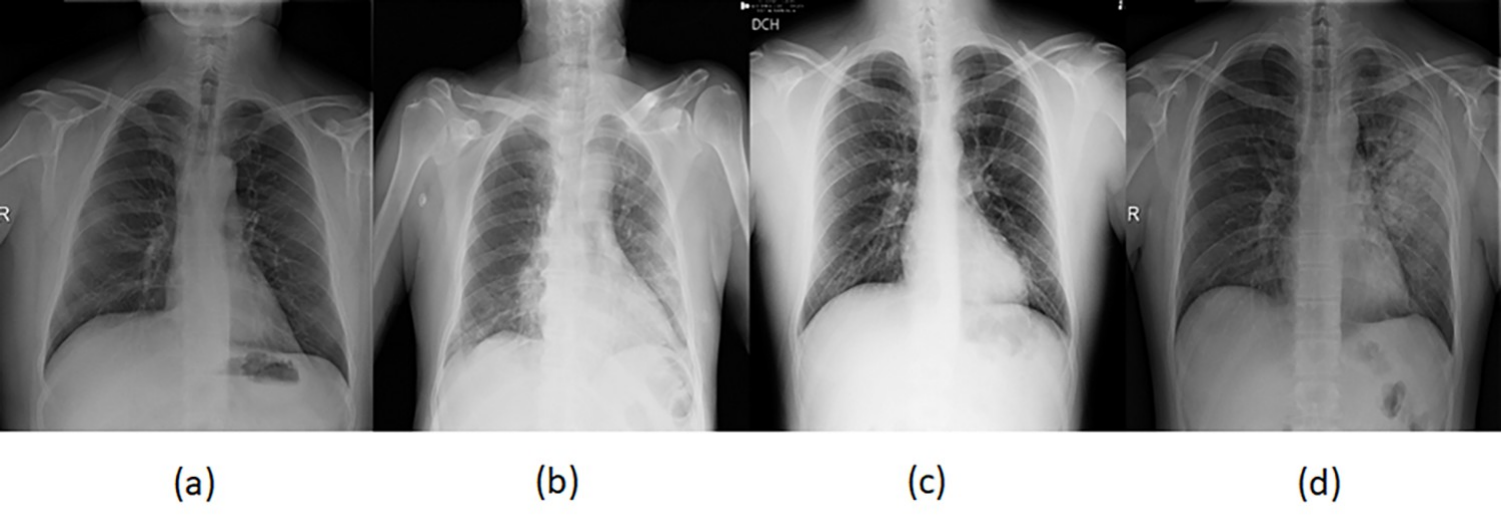
Sample images from the Kaggle [41] dataset (a) Negative for pneumonia (b) Typical (c) Indeterminate (d) Atypical.

SIIM-FISABIO-RSNA COVID-19 detection dataset was made available in the form of a public challenge at Kaggle [41]. The purpose of this dataset is the detection of COVID-19 and associated pneumonia types with subsequent localisation of lung opacity regions in the CXR images. The different classes of the CXR images are shown in Fig 1. The training dataset has a total of 6336 images of varying resolution ranging from 846x1353 to 4891x4020. The competition organizers provided the labels against the training dataset. The test dataset is divided into two portions: the public test dataset, which was used for computing the public Mean Average Precision (mAP) score before the end of the competition, and the private dataset, which was used to compute the final mAP score. The public test dataset consists of 1214 images while the complete dataset is around the same size as the training dataset. The number of the various image types in the training dataset is as shown in Table 1.

**Table 1:** Distribution of image classes.

Class	Number of Samples
Negative for Pneumonia	1737
Typical	3007
Indeterminate	1108
Atypical	484

### Proposed system architecture

**Fig 2:**
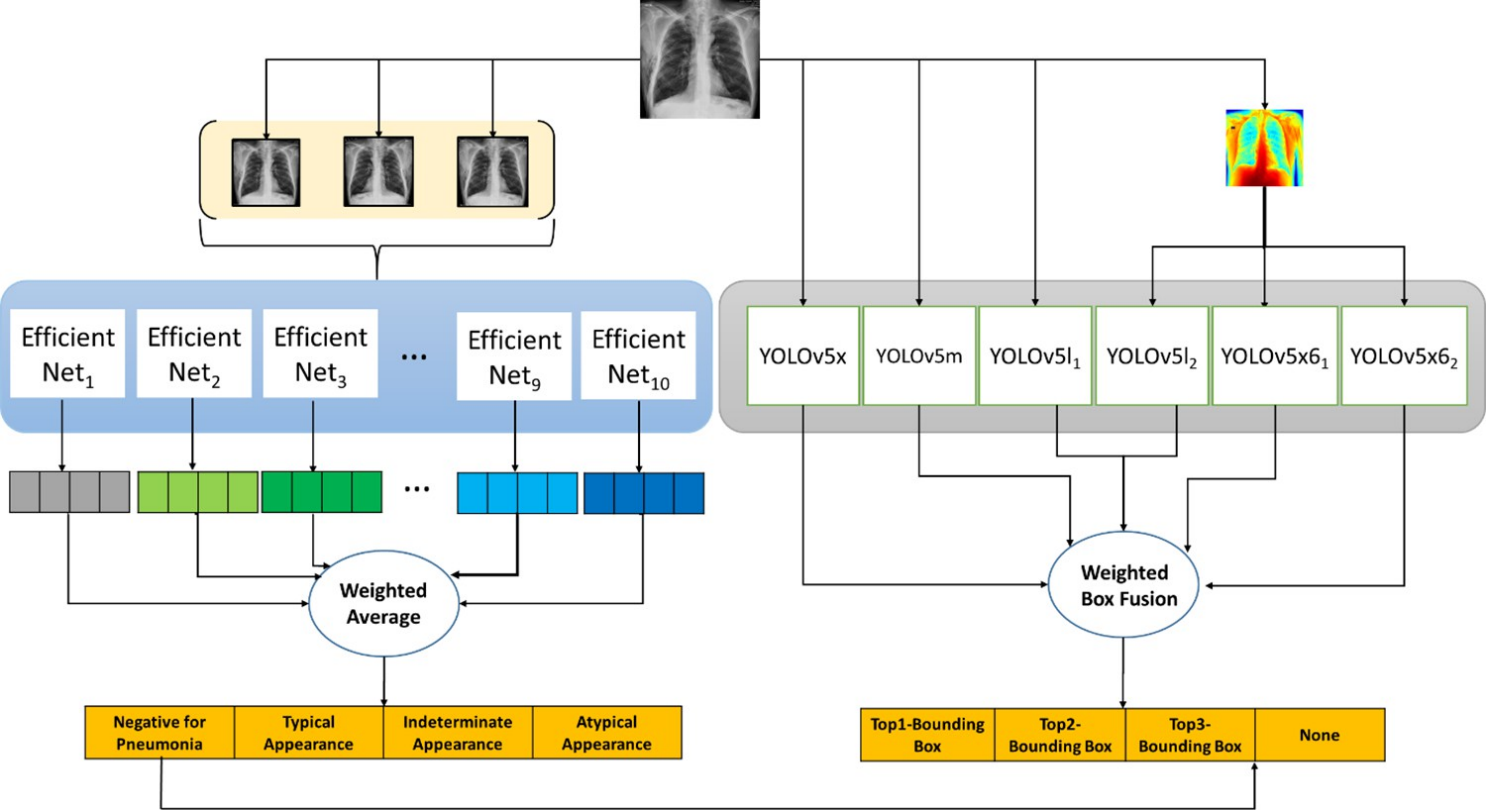
Proposed system architecture. Each EfficientNet_n_ (where n = 1, 2, 3 …, 10) has been trained on a different subset of train data. The variants of YOLOv5*a*_n_ (where n = 1, 2) have been trained in the same manner.

The proposed model for the classification of images is shown in Fig 2. We used the YOLOv5 [40] model for localizing the opacity and the EfficientNetv2-L model for grading the images into four classes—negative for pneumonia, typical appearance, indeterminate appearance and atypical appearance. An image of size 768x768 was provided as input which was then used for classification and of varying sizes for localizing opacity. The models were trained using TensorFlow 2.8 in Python on a system with 64 GB RAM and two Nvidia RTX 2070 GPUs. In order to train some models on higher image resolution, we also made use of Google Cloud using Google TPUs (v2.8).

In order to boost the performance of the models in the framework by generating more data, pre-processing and data augmentation techniques such as Min-Max normalisation and image flipping were performed. In contrast, Test Time Augmentation was performed by using only a subset of the augmentation techniques for the test images to get better performance.

#### Image pre-processing

This section describes the different techniques employed for image pre-processing and data augmentation for training both the classification and localisation models.

#### Pre-processing

The original dataset is provided in the Digital Imaging and Communications in Medicine (DICOM) file format in which the single channel pixel data is stored in 12 to 16 bits. Min-Max normalisation is performed on this pixel data and is then converted to an 8-bit unsigned integer. Furthermore, the single channel was replicated thrice to obtain a 3-channel (RGB) image that can be used as an input to the CNN.

One of the major limitations in deep learning is the trade-off between higher input size and more computational power required. In order to retain as much information as possible, the image must not be down sampled to a very low resolution. However, this raises the problem of computational cost. For the classification networks in the framework, the high-resolution images were resized to several sizes ranging from 380x380 to 768x768. The larger size of 768x768 provided the best performance with EfficientNetv2-L [16] and consequently all the models were trained using this image size. Similarly, a number of pre-processing techniques were used including unsharp masking with histogram equalisation, Contrast Limited Adaptive Histogram Equalisation (CLAHE) and Min-Max normalisation in the [0, 1] range.

Experimentally, unsharp masking with histogram equalisation resulted in slight performance improvements when smaller architectures like EfficientNetB4 [14] were used. CLAHE offered no discernible improvement when used with different CNN architectures. Min-Max normalisation in [0, 1] range was the only pre-processing technique used for the final models used in the framework because of its low computational overhead as compared to unsharp masking with histogram equalisation and better performance.

**Fig 3:**
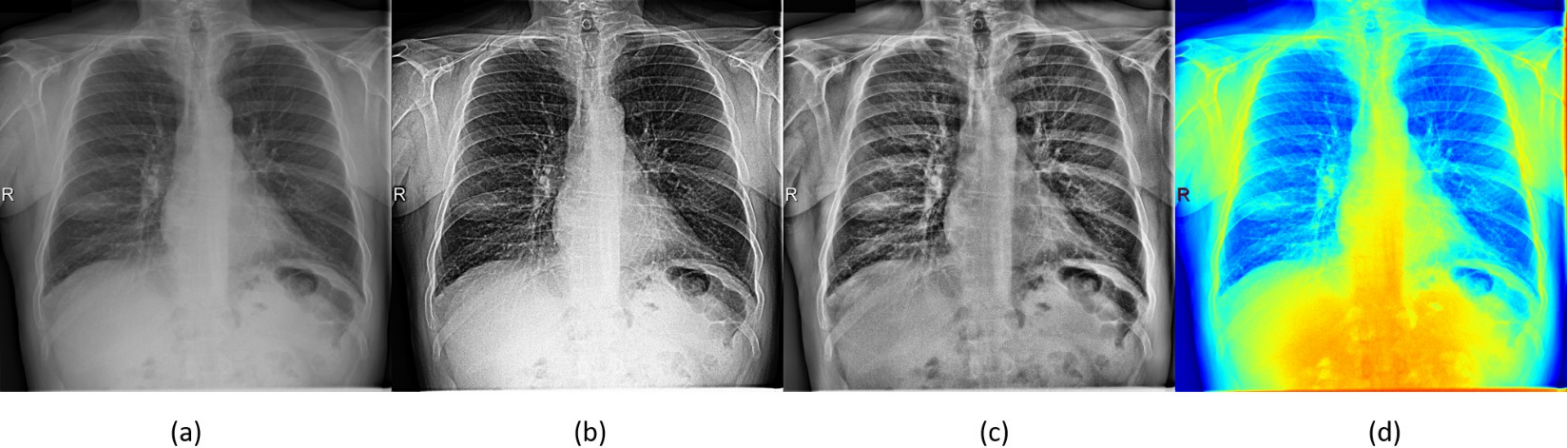
The original image (a) and the results of the selected image pre-processing techniques—(b) Unsharp Masking and Histogram Equalisation (c) CLAHE (d) Psuedo-Coloring.

For the localisation model ensemble, different image sizes were used to train the YOLOv5 [40] variants ranging from 640x640 to 1088x1088. For three models in the ensemble, the images were pseudo-coloured at different image sizes. Fig 3 shows the effect of different pre-processing techniques on a dataset image.

#### Data augmentation

The performance of CNN models to a great extent is attributed to massive labelled data which is difficult for medical imaging applications [4]. The class imbalances in medical imaging applications can be addressed using data augmentation i.e. using random transformations to increase the dataset with common techniques such as resizing, warping, lighting, flipping etc. [32].

**Fig 4:**
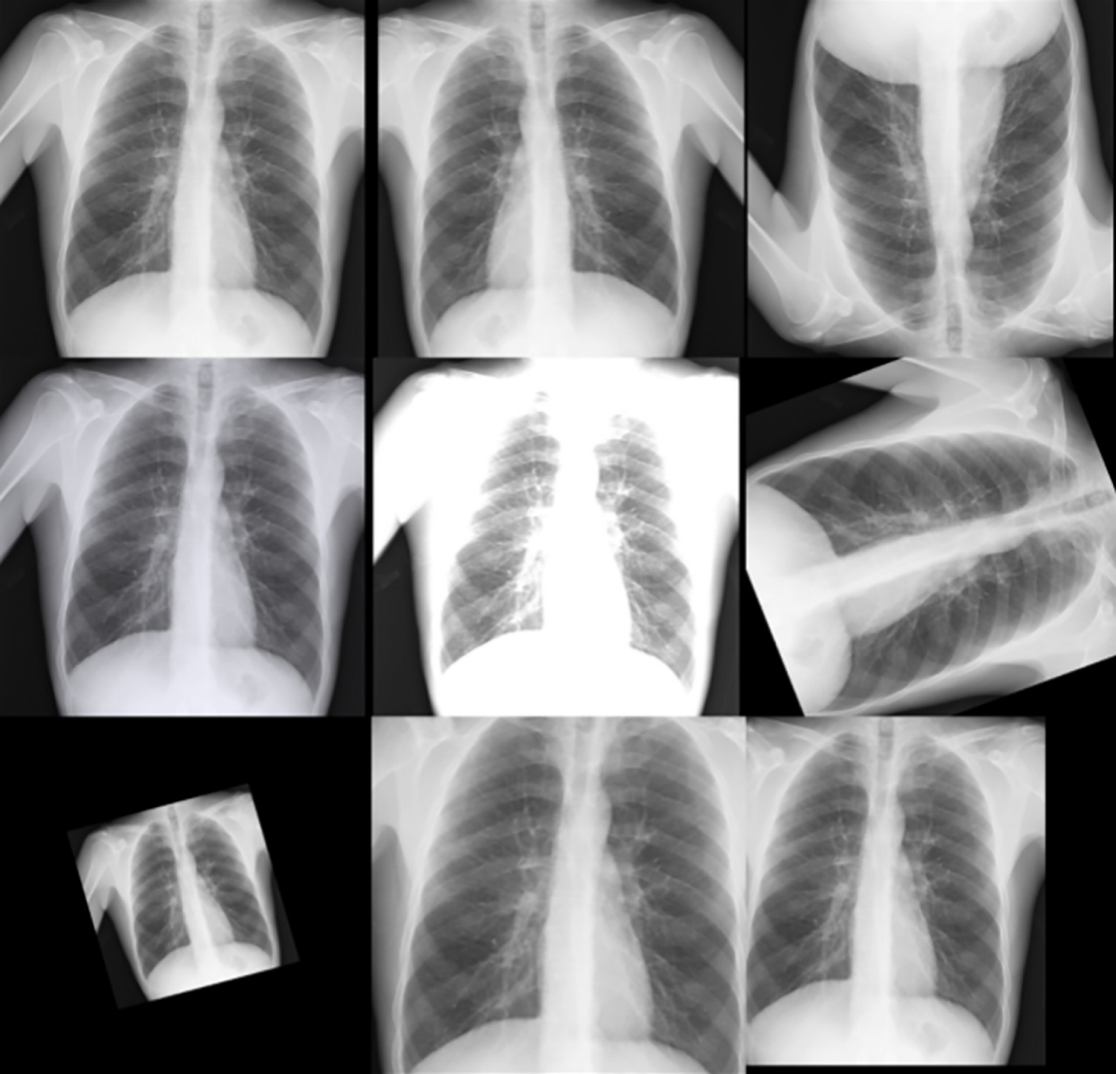
Image Augmentation [from Top to Bottom] Original, Horizontal Flipped, Vertical Flipped, Saturation, Contrast, Rotation, Shear, Zoom and Shift.

As the SIIM dataset [41] has imbalanced classes, data augmentation can help alleviate this problem to some extent and can help train the CNN better due to added variation in the dataset. Keeping this view, multiple data augmentation techniques were used for training both the classification and localisation models which included: flipping (left to right and up to down), random saturation, random brightness, random contrast, random rotation, random shear, random zoom and random shift. The effects of these operations performed for data augmentation are shown in Fig 4. In addition to the above-mentioned data augmentation techniques, a few other techniques such as RGBShift, Random Flare, Random Fog and Random Snow were also tested. However, these were dropped because these did not provide any improvement.

**Fig 5:**
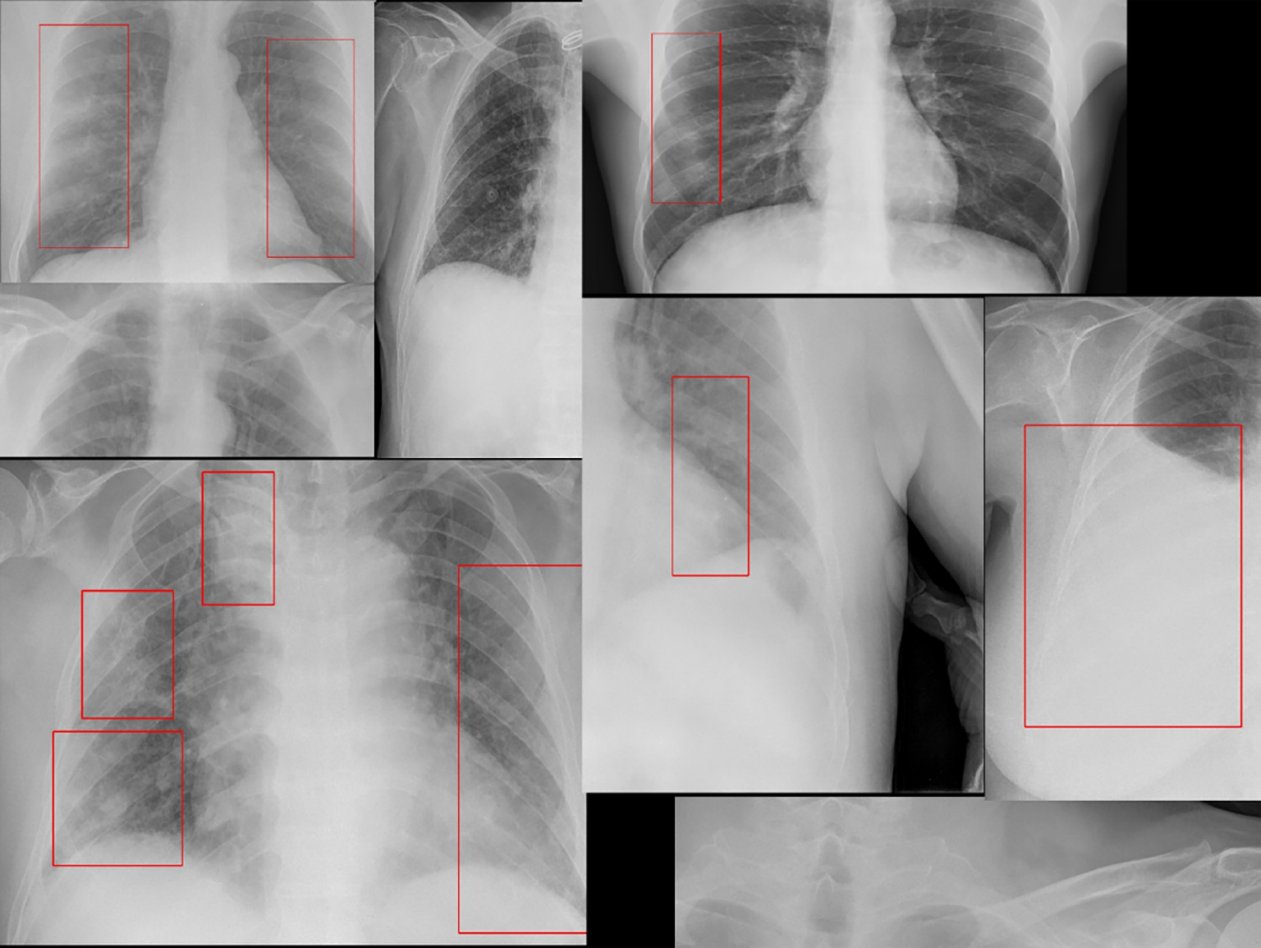
Region of interests for opacity localisation.

For localisation networks (see Fig 5), the above-mentioned augmentation techniques were used along with the mosaic image augmentation provided in YOLOv5 [40]. The mosaic image augmentation technique increases the number of Region-of-Interests in a single image.

### Proposed CNN model

The CNN model performance, to a large extent, depends on the data quality and the choice of model hyperparameters. These models have shown exemplary performance for image classification, segmentation, and detection tasks [11]. A value or weight automatically learned during the model training is termed a parameter, whereas a hyperparameter is a value that needs to be set before the training begins [12]. Innovations in CNN are parameter and hyperparameter optimization, modification of processing units and layer connectivity etc. [11].

#### Image classification with EfficientNetv2-L

**Fig 6:**
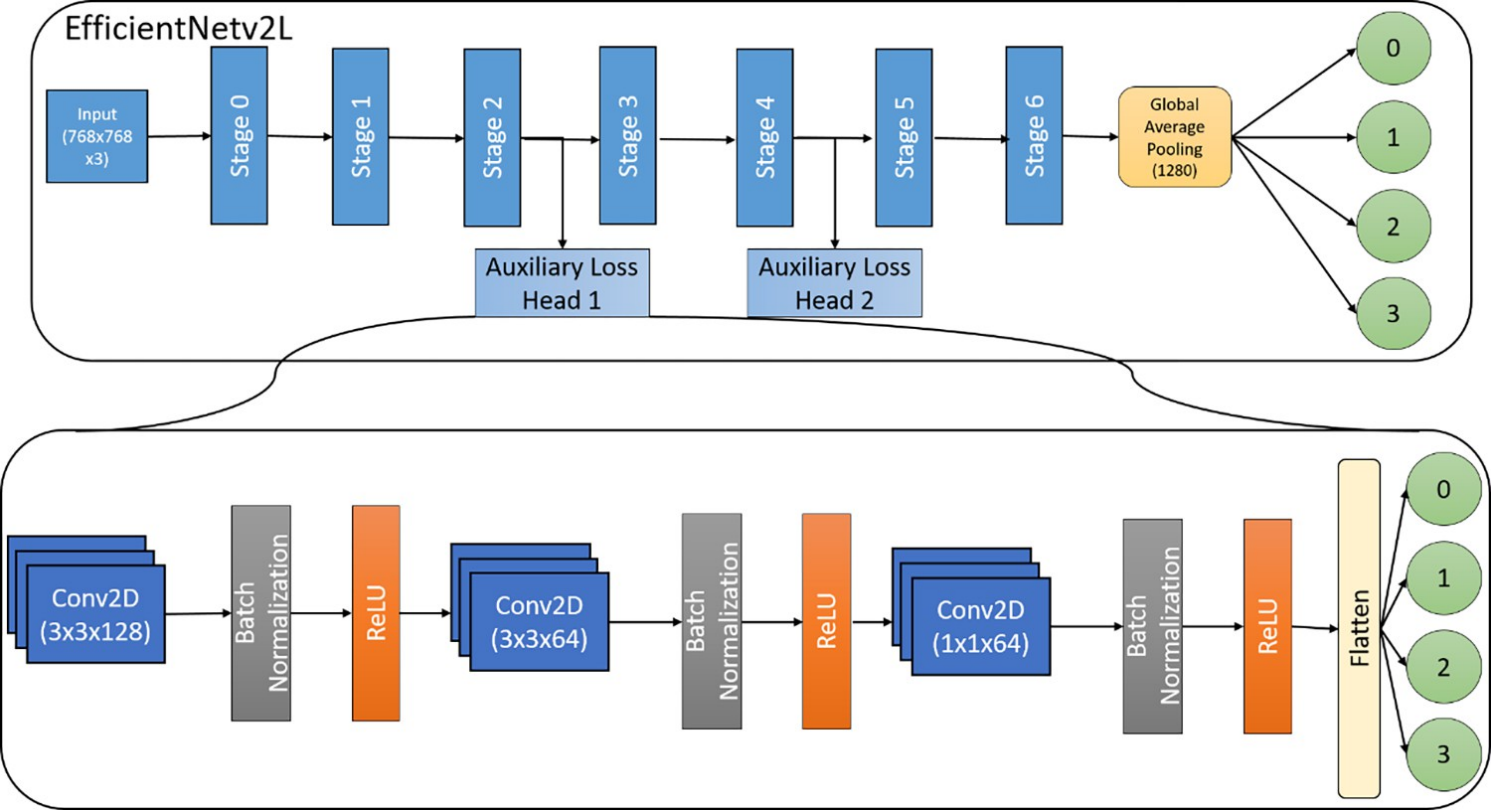
Proposed neural network model.

We used the CNN model EfficientNetv2-L [16] for the classification. TensorFlow along with Keras [42] and TensorFlow Hub [43] was used for training the EfficientNetv2-L [16]. The proposed model is shown in Fig 6.

In order to ensure that the trained models generalised despite the class imbalance, two auxiliary heads were added to the model. These auxiliary heads functioned as classification heads for the same four classes as the final output. The auxiliary heads consisted of a four-layer CNN with initial three layers being convolutional layers while the last one being a dense layer. The simplicity of this auxiliary head architecture allowed for minimal training overhead. The weights for each auxiliary head were set to 0.2 while the final classification heads’ contribution was 0.6. Addition of the auxiliary heads improved the performance on the public test dataset.

A 5-fold cross validation technique was used to train the EfficientNetv2-L model. In this approach, for each instance of the model, only 20% of the training dataset was used. This technique has the benefit of having several different trained models on different subsets that may have slightly different distribution of data.

#### Opacity localisation with YOLOv5

YOLO (You Only Look Once) has a CNN backbone for feature extraction and localisation and is used for real-time object detection. The models are pre-trained on the COCO dataset [40]. In comparison with the earlier object detection models it is much faster and provides better performance.

#### Hyperparameter optimisation

Hyperparameters are the parameters that define the model and must be selected and set before the model training. Hyperparameters need to be optimised for better results and different methods can be used [37, 44]. Test Time Augmentation along with Keras [42] and TensorFlow Hub [43] was used for training the EfficientNetv2-L. Instead of initialising the weights randomly, the pre-trained weights of ImageNet21K were used which have been further fine-tuned on ImageNet21K. The hyperparameter values are shown in Table 2.

**Table 2:** Hyperparameter values used in proposed architecture.

Hyperparameter	Classification Models	Localisation Models
Learning Rate	0.001	0.01
Loss Function	Categorical Cross entropy	Binary Cross Entropy with Logit Loss
Batch Size	64	8
Optimisation	Adam	SGD
Parameters (Million)	117.8	21.2–140.7

In addition to the above mentioned finalised hyperparameters, a number of other hyperparameters were tested including Binary and Focal Loss for classification models and categorical cross entropy for localisation. However, these variations to the hyperparameters did not improve the results. The framework’s behaviour at the time of inference can be summarised in algorithmic form as shown in Algorithm 1.

    **Algorithm 1. Algorithmic form of inference for the proposed framework**.

**Input:** Grayscale Chest X-Ray (CXR) Image

    Classification Models (all models retained in memory 1 ~ M)

    Localisation Models (all models retained in memory 1 ~ L)

    Test Time Augmentations (all augmentations retained in memory 1 ~ T)

    Weights for Each Model

    Input Size for Each Model

**Output:** Class probability for 4 classes, top *n* bounding boxes

1 Convert single channel CXR to 3 channel

2 Resize CXR to size 768x768

3 Create a final vector to store final probabilities for each class

4 **for** *i* = 1 to M

5    Create an empty temporary vector to store probabilities for each class for each Test Time Augmentation

6    **for** j = 1 to T

7        Apply augmentation j on image

8        Perform inference on image using model i and add to temporary vector

9    Average the values in the temporary vector

10    Add the average value from the temporary vector to the final vector

11 Compute weighted mean from the final vector

12 Create a final list to store final bounding boxes for each image

13 **for** k = 1 to L

14    Resize CXR to appropriate size for model input

15    **if** model requires pseudo-color input

16        Apply pseudo-color

17    Create an empty temporary list to store bounding boxes for each image for each Test Time Augmentation

18    for o = 1 to T

19        Apply augmentation j on image

20        Perform inference on image using model k and store results in temporary list

21    Perform Non-Maxima Suppression to combine overlapping bounding boxes

22 Apply Weighted Box Fusion on the boxes obtained by loclaisation models

23 **Return** the final class probability vector and top n bounding boxes

### Performance metrics

The model performance can be determined by combining TP (True Positive), TN (True Negative), FP (False Positive), and FN (False Negative). These measures are derived from the relationship between the actual and predicted values of true and false instances of a class in a classification system and are given below from Eqs (1) to (4).


$$Accuracy=\left(\frac{TP+TN}{\left(TP+TN+FP+FN\right)}\right)$$ (1)

$$Precision=\left(\frac{TP}{\left(TP+FP\right)}\right)$$ (2)

$$Recall=\left(\frac{TP}{\left(TP+FN\right)}\right)$$ (3)

$$F1=\left(\frac{Precision*Recall}{\left(Precision+Recall\right)}\right)$$ (4)

Classification accuracy of a model is the ratio of correctly predicted instances and total instances. However, in case of class imbalance, accuracy may not be sufficient on its own. Precision or specificity is the ratio of correctly predicted positive instances and the total instances predicted as positive. Similarly, recall and sensitivity defines the ratio of correctly predicted positive instances and the total actual positive instances. Recall is the ability of a classifier to determine all the true instances per class. F1 is the harmonic mean of precision and recall and indicates a balance between precision and the recall.

Mean Average Precision (mAP) is the mean taken over per class Average Precision [45] and is a commonly used metric for image classification competitions.

## Results

In order to gauge the performance of the classification and localisation ensembles, the results have been computed on both the training dataset and the test dataset. This approach was taken as the labels for the SIIM test dataset [41] have not yet been made public. Therefore, in order to look at the detailed performance of the classifiers, the training dataset was also used for computation of performance metrics. The metrics for the test dataset have also been reported but they are limited to the metrics that were computed by the organisers of the competition for each solution.

One thing that should be noted is that in order to compute the results on the test dataset, there was a limitation that the output file with the labels and the annotations should be in a pre-specified format. This meant that for computing the results for the classification and localisation modules of the framework, the irrelevant portion of the submission file had to be brought back to the original state. So, while the mAP score predominantly came from the module that was being tested, the original state of the other module still played a role. However, this component of the mAP score was constant for comparison between all the different iterations of a module, thus providing a level field to ascertain the performance of different classifiers and localisers.

### Multiclass classification

As mentioned earlier, the dataset was split into 5 folds with each fold used to train a separate classifier. This 5-fold split was repeated twice resulting in 10 different models. The Out of Fold (OoF) data, i.e. the data that was not used for training that model, was used to calculate the metrics for each trained classifier as shown in Table 3.

**Table 3:** Model performance metrics.

Fold	Negative for Pneumonia			Typical			Indeterminate			Atypical		
	Precision	Recall	F1 Score	Precision	Recall	F1 Score	Precision	Recall	F1 Score	Precision	Recall	F1 Score
1	0.7	0.88	0.78	0.72	0.91	0.8	0.58	0.004	0.007	0.56	0.29	0.32
2	0.69	0.91	0.78	0.74	0.89	0.81	0.47	0.09	0.15	0.58	0.25	0.35
3	0.69	0.91	0.79	0.75	0.89	0.81	0.51	0.11	0.18	0.61	0.3	0.4
4	0.69	0.88	0.78	0.75	0.87	0.81	0.44	0.12	0.18	0.44	0.32	0.37
5	0.65	0.89	0.75	0.72	0.9	0.8	0.65	0.03	0.07	0.59	0.2	0.3
6	0.68	0.89	0.77	0.74	0.88	0.81	0.55	0.04	0.07	0.5	0.41	0.45
7	0.7	0.89	0.78	0.72	0.89	0.8	0.5	0.06	0.1	0.51	0.32	0.39
8	0.71	0.87	0.78	0.72	0.91	0.8	0.56	0.05	0.09	0.53	0.36	0.43
9	0.74	0.87	0.8	0.74	0.9	0.81	0.52	0.17	0.26	0.57	0.35	0.43
10	0.69	0.88	0.78	0.74	0.9	0.81	0.54	0.12	0.2	0.53	0.24	0.33

The combined confusion matrix for all the trained models is shown in Table 4. It shows the distribution of predicted classes in four outputs. The mAP values for each fold and the ensemble are shown in Table 5.

**Table 4:** Combined confusion matrix.

Class	Negative for Pneumonia	Typical	Indeterminate	Atypical	Accuracy
Negative for Pneumonia	1233	128	13	15	0.887689
Typical	195	2149	27	35	0.893184
Indeterminate	265	495	73	53	0.082393
Atypical	82	157	30	117	0.303109
Accuracy					0.704954

**Table 5:** Mean average precision.

Fold	Mean Average Precision (mAP)	
	Public Test	Private Test
1	0.434	0.416
2	0.433	0.421
3	0.437	0.420
4	0.43	0.414
5	0.43	0.408
6	0.422	0.408
7	0.431	0.416
8	0.424	0.415
9	0.431	0.415
10	0.436	0.414
Ensemble	**0.444**	**0.427**

Along with the multiclass classification for the images, a confidence score for the absence of opacities in an image was also required in the localisation module. This score was computed by simply taking the average of the negative class score for an image from all the classifiers.

### Opacity localisation

**Fig 7:**
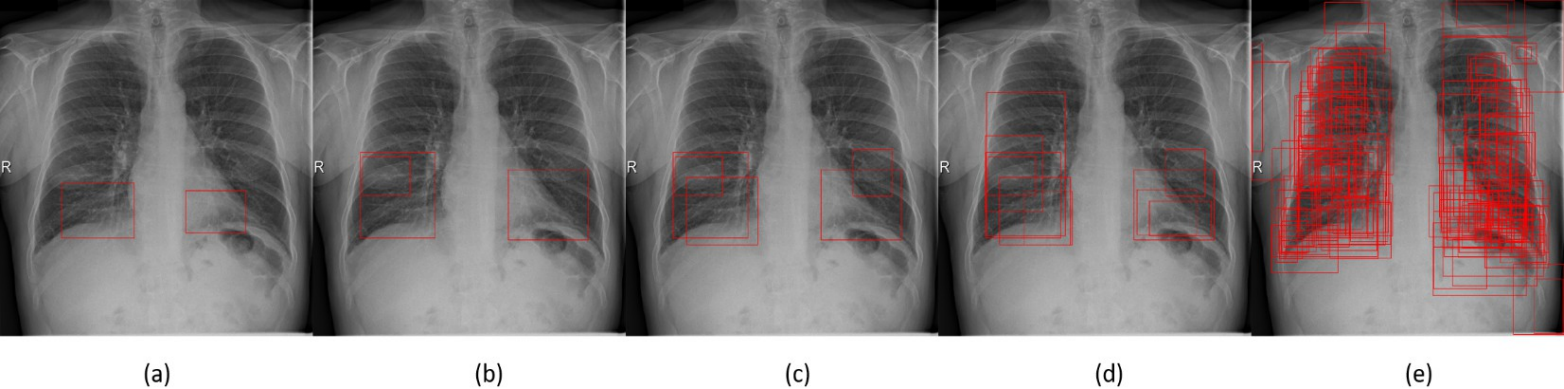
Results of opacity localisation: (a) Ground Truth (b) WBF Top 3 Bounding Boxes (c) WBF Top 5 Bounding Boxes (d) WBF Top 10 Bounding Boxes (e) WBF All Bounding Boxes with lowered threshold.

As is the case with class labels, the bounding box annotations for SIIM test dataset [41] are also absent. Therefore, using the same methodology that was used for computation of classification results, the mean average precision (mAP) has been calculated for the training dataset. In order to increase the mAP, the number of bounding boxes per image can be increased. As the bounding boxes are sorted by their confidence score before mAP is computed, therefore having excess bounding boxes can only improve the score even though its effect might be minute. However, restricting the bounding boxes to just three also results in correct opacity detections. Fig 7 shows the effect of varying the number of bounding boxes in comparison to ground truth with three bounding boxes being the closest to the ground truth.

Table 6 shows the mAP score that has been computed for the training dataset along with the mAP score for public and private datasets. While the individual models performed relatively close to one another, the improvement in performance was a result of Weighted Boxes Fusion [46] which ensembles the bounding box detections from all the individual models.

**Table 6:** Mean average precision for the training dataset.

Model	Mean Average Precision (mAP)				
	Training Data			Public Test	Private Test
YOLOv5x	**0.7086**			0.132	0.093
YOLOv5x6 (Fold 1)	0.612			0.138	0.093
YOLOv5l (Fold 1)	0.6748			0.137	0.093
YOLOv5l (Fold 2)	0.6099			0.137	0.093
YOLOv5x6 (Fold 2)	0.5932			0.137	0.093
YOLOv5m	0.6212			0.142	0.094
Weighted Boxes Fusion	0.6981			**0.147**	**0.143**
	0.6384	0.6736	0.69		
	Top 3 Bounding Boxes	Top 5 Bounding Boxes	Top 10 Bounding Boxes		

### Comparison with other methodologies

SIIM-FISABIO-RSNA COVID-19 Detection competition hosted by Kaggle [41] provided an opportunity to explore some of the other methodologies employed for solving the classification and localisation problem for the same dataset. Some of those techniques were quite similar to our proposed methodology while others differed significantly. Consequently, these techniques had varying results. The comparative results of the proposed framework with top scoring methodologies by other researchers are shown in Table 7.

**Table 7:** Comparison with top scoring methodologies on [41].

Item	Technique	mAP	
		Public Test	Private Test
1	Use of multiple external training datasets including NIH and ChexPert for training the model ensembles with auxiliary loss before fine tuning them on the competition dataset. All models were trained on an ROI containing just the lungs.	**0.658**	**0.635**
2	Pre-training of models using external datasets including NIH and BIMCV for all the models with auxiliary loss for classification and localisation	0.645	0.634
3	Pre-training of transformer models with auxiliary heads using external datasets including ChexPert and VinBigData for both classification and localisation.	0.654	0.631
4	Pre-training using external NIH dataset for models with segmentation auxiliary heads using lung segmentation masks	0.649	0.628
5	Due to mutual exclusivity of the classes, training of a single localiser for both the opacity localisation and classification task using 1-pixel wide bounding boxes for classification tasks	0.639	0.624
6	Training of models with auxiliary heads for classification and localiser training using pseudo-coloured images **(Proposed Framework)**	0.617	0.609

It is evident from Table 7 that pre-training on various data sets is a methodology that is utilised by many of the other researchers and has become commonplace, particularly for CXR [39, 47]. This enables the trained models to understand how the characteristics in CXRs are represented at the local level. Better models are then produced for newer, untested datasets using the previously learned information in the form of pre-trained weights. A further benefit of this strategy is that the models can be used widely due to their improved generalizability. Another approach has been to model the problem as a purely localisation problem where the classification classes are combined with opacity class. This allows the network to be able to distinguish between the representations of different diseases at pixel level resulting in a better classification accuracy. The comparison also highlights that the proposed approach has comparable results with other approaches using test time augmentation for classification and localization along with auxiliary heads without large scale pre-training.

However, in order to gauge the performance of our methodology on another dataset, RSNA Pneumonia Detection Challenge [48] was used which has been used by [49, 50]. [48] poses a similar problem as [41] and therefore our proposed methodology can be used here as well to localize opacities in the pneumonia images. The results are presented in Table 8.

**Table 8:** Comparison of proposed methodology with existing techniques on RSNA dataset.

Methodology	Stage 2		
[49]	Retina Net	Mask RCNN	Combined
	0.202	0.165	0.204
[50]	Mask RCNN (ResNet50)	Mask RCNN (ResNet101)	Combined
	0.183	0.199	0.218
Proposed	0.175		

It must be noted here that the results achieved by our proposed methodology are without any fine tuning or retraining on RSNA data set [48]. Even without any retraining, we were able to achieve reasonable performance on a completely unseen data set. This performance could be improved further by fine tuning the localization models on RSNA data set [48] and re-training the classification models using the same data as well.

## Discussion

In order to achieve the best performance, several frameworks with different CNN architectures were tested along with the proposed framework. The choice of going with a deeper and large network like the EfficientNetv2-L rather than ResNet50 also stems from the fact that the inter-class variation for this dataset is relatively low. Therefore, more parameters usually mean better results. In addition, EfficientNetv2-L incorporates architecture level changes such as new base operations which make it better than other models. Some other observations that arose from this were:

- CNN architectures with more trainable parameters did not necessarily offer better results.
- Auxiliary heads incorporated in the CNN architectures in the earlier stages offered a considerable improvement as compared to models with no auxiliary heads.
- For classification models, image size had a negligent effect on the performance.
- Localisation models performed best when a different combination of input image sizes was used.

While the overall performance of the classification ensemble is reasonable, the Indeterminate and Atypical class are the worst performing classes. One of the reasons is that the vast majority of the training dataset is split between the first two classes. This is the same reason why pre-training with different publicly available dataset has resulted in not only overall better performance of the models but has resulted in better classification performance for the aforementioned classes. In short, the poor performance for our classification ensembles for the Indeterminate and Atypical class can be attributed to the lack of pre-training on publicly available datasets.

Although deep learning architectures achieve commendable performance in medical image classifications, why a particular prediction was made is not clear as the models have a black-box nature [4]. Explainability is thus one of the key problems to be solved [4] before the models can be trusted. An issue in ML research is the lack of high-quality training data in sufficient numbers [4].

**Fig 8:**
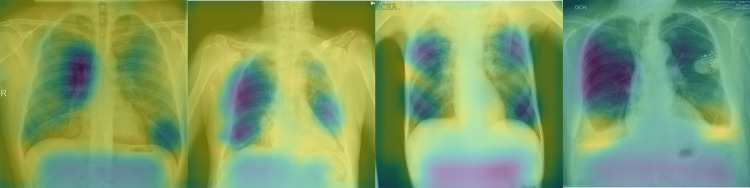
Gradient-weighted Class Activation Mapping (Grad-CAM): From left to right–Negative for pneumonia, typical appearance, indeterminate and atypical appearance. The heat map of the activations show that the area of high activations shrinks in diseased images as compared to healthy images.

Gradient-weighted Class Activation Mappings (Grad-CAM) can shed light on the features that the network pays importance to for making its decision. Using Grad-CAM with our trained model and the images from the four classes, it is evident (Fig 8) that the network is able to distinguish between healthy lungs (absence of pneumonia) and diseased lungs with varying degrees of disease.

Most of the techniques employed for classification and localisation on the dataset relied on ensembles of varying sizes with multiple state-of-the-art CNN architectures trained using different subsets of the training data and initialised using readily available, significantly large datasets of CXR images such as NIH. Since there is no pre-training involved, our suggested methodology is computationally cheap and takes minimal training time. In addition, the performance was further improved by adding auxiliary heads at several places along the CNN architecture. Even though auxiliary heads have been surpassed in favour of deeper and wider architectures, they played an important role for this particular problem, as the class sample mismatch was significant. Furthermore, the auxiliary heads forced the trained networks to generalise better; this approach was necessary as only a fraction of the total test data was available for computing the mAP that was the indicator being used for selecting the overall best frameworks. Although the issue is mitigated by the inclusion of auxiliary heads, because we have not pre-trained our models on other publicly accessible datasets, their generalizability may deteriorate when applied to datasets that have never been seen before.

As opposed to a single model that has been trained at several input image sizes, an ensemble can perform better when used for opacity localisation at various image sizes. Using an ensemble of many models for localization and classification can be detrimental to inference.

## Conclusion

The diagnosis of COVID-19 is critical in the early stages of the infection and one reliable mechanism for disease diagnosis is by using chest X-ray (CXR) images which are readily acquired and commonly accessible compared to other image modalities such as Computed Tomography (CT). This paper proposes the use of Convolutional Neural Network (CNN) architecture, EfficientNetv2-L for multi-classification of CXR images into COVID-19, pneumonia, normal and atypical classes on the Kaggle dataset [41]. We provide results for class wise accuracy, sensitivity and specificity and conclude that an ensemble of models is a promising technique for accurate classification of CXR images. Explainability of images is a recent trend in deep learning image diagnosis research [51]. This had not been a problem with earlier rule-based Machine Learning models where it was easier to understand why a particular prediction was made [51]. The trust in Deep Learning models can be enhanced by identifying the salient areas in CXR images that led to a prediction [51]. Similarly, an estimate of confidence with a prediction could be helpful and not making a prediction in case of low confidence [51]. The majority of Machine Learning application for medical applications are in radiology using supervised learning [51]. The improvement in healthcare AI has been demonstrated by many studies but the clinical value is yet to be realised [32, 51].
